# How severe would prioritization-induced bottlenecks need to be offset the benefits from prioritizing COVID-19 vaccination to those most at risk in New York City?

**DOI:** 10.1186/s12889-022-14846-7

**Published:** 2023-01-26

**Authors:** Hae-Young Kim, Anna Bershteyn, Jessica B. McGillen, R. Scott Braithwaite

**Affiliations:** Department of Population Health, New York University Grossman School of Medicine, 227 E 30th Street, NY 10016 New York, USA

**Keywords:** COVID-19, Vaccination, Prioritization, Variant

## Abstract

**Background:**

Prioritization of higher-risk people for COVID-19 vaccination could prevent more deaths, but could slow vaccination speed. We used mathematical modeling to examine the trade-off between vaccination speed and prioritization for individuals age 65+ and essential workers.

**Methods:**

We used a stochastic, discrete-time susceptible-exposed-infected-recovered (SEIR) model with age- and comorbidity-adjusted COVID-19 outcomes (infections, hospitalizations, and deaths). The model was calibrated to COVID-19 hospitalizations, ICU census, and deaths in NYC. We assumed 10,000 vaccinations per day, initially restricted to healthcare workers and nursing home populations, and subsequently expanded to other populations at alternative times (4, 5, or 6 weeks after vaccine launch) and speeds (20,000, 50,000, 100,000, or 150,000 vaccinations per day), as well as prioritization options (+/− prioritization of people age 65+ and essential workers). In sensitivity analyses, we examined the effect of a SARS-COV-2 variant with greater transmissibility.

**Results:**

To be beneficial, prioritization must not create a bottleneck that decreases vaccination speed by > 50% without a more transmissible variant, or by > 33% with the emergence of the more transmissible variant. More specifically, prioritizing people age 65+ and essential workers increased the number of lives saved per vaccine dose delivered: 3000 deaths could be averted by delivering 83,000 vaccinations per day without prioritization or 50,000 vaccinations per day with prioritization. Other tradeoffs involve vaccination speed and timing. Compared to the slowest-examined vaccination speed of 20,000 vaccinations per day, achieving the fastest-examined vaccination speed of 150,000 vaccinations per day would avert additional 313,700 (28.6%) infections and 1693 (24.1%) deaths. Emergence of a more transmissible variant would double COVID-19 infections, hospitalizations, and deaths over the first 6 months of vaccination. The fastest-examined vaccination speed could only offset the harm of the more transmissible variant if achieved within 5 weeks of vaccine launch.

**Conclusions:**

Faster vaccination speed with sooner vaccination expansion would save more lives. Prioritization of COVID-19 vaccines to higher-risk populations would be more beneficial only if it does not create an excessive vaccine delivery bottleneck.

**Supplementary Information:**

The online version contains supplementary material available at 10.1186/s12889-022-14846-7.

## Introduction

In the winter of 2020, New York City (NYC) faced a coalescence of two major developments in severe acute respiratory syndrome coronavirus 2 (SARS-CoV-2) pandemic: the availability of coronavirus disease 2019 (COVID-19) vaccines and the identification of new variants of SARS-CoV-2. COVID-19 disproportionately affects older people and those with underlying conditions as well as people at a higher risk of exposure [1–3]. When COVID-19 vaccines became available in mid-December 2020 initially in limited quantities, vaccines were first offered to healthcare workers and nursing home populations [4, 5]. Eligibility expanded in January 2021 to include age 65 or older and essential workers (first responders and healthcare, transit, education, and public safety workers), and later to other groups [5, 6].

While prioritization of higher-risk people could prevent more deaths by ensuring vaccines are delivered to priority populations before being offered to others, prioritization could also slow vaccination speed due to bottlenecks associated with reaching and verifying vaccine-eligible individuals. Concerns have been raised in NYC and elsewhere about bottlenecks in vaccine delivery, as evidenced by a low proportion of stocked vaccines that had been administered to patients in December 2020 and early January 2021 [7]. Some hypothesized that removing prioritization for high-risk populations could reduce these bottlenecks and increase vaccination speed [8].

Contemporaneously with vaccine launch, SARS-CoV-2 variants of concern were reported in Europe, Africa, and South America. The lineage B.1.1.7 was first detected in the United Kingdom (UK), where it grew from a rare variant to the dominant circulating variant [9]. In NYC, the first two cases of B.1.1.7 were confirmed at the end of December 2020. Based on its rate of growth in the UK, B.1.1.7 was estimated to be more infectious than previously dominant variants by a factor of 56% (95% CI − 8 to + 128%) [10]. The emergence of a more transmissible variant could have important implications for the role of vaccination in combatting COVID-19 in NYC. Specifically, prioritization bottlenecks could be more harmful in the context of a more transmissible variant because of lost opportunities to slow epidemic growth.

We hypothesized that (i) there is a trade-off between prioritization and vaccination speed (i.e., the number of vaccinations delivered per day), and (ii) this trade-off varies based on whether or not a more transmissible variant emerges in NYC. We used a mathematical model to estimate the impact of vaccination on the COVID-19 epidemic under different timings of vaccination expansion and speed, with and without the emergence of a more transmissible variant in NYC. We then assessed the maximum prioritization bottleneck under which prioritization of high-risk groups would still avert more deaths than vaccination without prioritization.

## Methods

### Mathematical model

We augmented a stochastic, discrete-time susceptible-exposed-infected-recovered (SEIR) model of SARS-CoV-2 transmission [11–13] with age- and comorbidity-adjusted COVID-19 outcomes (symptoms, hospitalization, ICU admission, and death) using data on NYC’s distribution of age and chronic conditions from New York Behavioral Risk Factor Surveillance System Data in 2017 [14], the 2013–2014 New York City Health and Nutrition Examination Survey (NYC HANES) [15], and the US census [16]. We included lagged transmission of community-acquired infections to household members and assumed a secondary attack rate of 25% for within-household transmission [17].

We assumed an average time from infection to symptom onset of 5.1 days [18], and an average from symptom onset to hospitalization of 11 days [19]. We assumed that symptomatic infections were tested and diagnosed as COVID-19 cases. The length of hospitalization, ICU stays, hospitalization rate, and the mortality rate among the hospitalized were assumed to be time-varying, based on the information and the publicly available data provided by NYC Department of Health and Mental Hygiene (DOHMH) from April 2020 to December 2020 [20]. The hospitalization rate declined from 26.6 to 9.5% over this time period based on empirically observed improvements in treatment together with changes in the age distribution of hospitalized persons, and we used a sigmoid function to approximate the time-course of this decline. The average lengths of stay at hospitals among non-intensive care unit (ICU) admitted patients were assumed to be 5–11 days. Critically ill patients were assumed to be first admitted to non-ICU hospital beds for 3 days, then transferred to the ICU for 6–21 days before returning to non-ICU hospital beds for another 1–14 days. All model input parameters and assumptions are described in detail in the supplement.

We calibrated the model to publicly available data on daily new hospitalizations for COVID-19 and confirmed and probable COVID-19 attributable deaths from the NYC DOHMH [20], and the internal data on the daily number of patients with COVID-19 in ICUs from the NYS Department of Health’s Hospital Emergency Response Data System (HERDS). Cases included confirmed and probable cases as defined by the US Centers for Disease Control and Prevention (CDC) [21, 22]. Death was classified as confirmed if the decedent had a positive molecular test for COVID-19 and did not die of apparent external causes such as gunshot wounds, and as probable if the decedent had no known positive molecular test for COVID-19 but had COVID-19 as a cause of death in the death certificate [20]. Diminution in the basic reproduction number (R_0_) due to mask-wearing and social distancing was benchmarked based on the observed growth rate in detected cases in early January 2021 after adjustment for percent positivity. We extrapolated R_0_ forward with a linear increase in R_0_ up to 56% over 6 weeks from January 15th to represent the emergence of a more transmissible variant. The period of 6 weeks was chosen based on the rapid growth rate of the linkage B.1.1.7 observed in the UK, where the percentage of detected cases with lineage B.1.1.7 in London and East of England increased from < 10% in mid-November to almost 100% by the end of December 2020 [9]. The model was implemented in Python 3.7 and outputs were analyzed and graphed using R 3.6.1.

### Assumptions about vaccination

Numbers of vaccinations were defined as a full vaccine course, regardless of the total number of doses required. Vaccine efficacy was assumed to reach 95% starting 11 days after vaccination [23] and last at least 6.5 months (from the earliest dose delivered on December 15th, 2020, until the end of the period of analysis on July 1st, 2021). For vaccines requiring multiple doses, the vaccination speeds reported are for the first dose only. We assumed no waning of immunity from vaccination or natural infection. Based on the prior vaccination coverage of influenza among HCW in the U.S. [24], willingness to be vaccinated was assumed to be 90% for healthcare workers (HCW) and 70% for non-HCW, with lower rates explored in sensitivity analyses.

We assumed three groups for prioritization: (1) HCW and nursing home residents; (2) people age 65+ and essential workers; and (3) all other people. These priority groups are a simplification of the four-phase rollout-plan announced by the CDC with two of the phases collapsed: Phase 1a) HCW and nursing home residents; Phase 1b) essential workers and people aged 75+ years; Phase 1c) people aged 65+ years and people aged 16–64 years with underlying medical conditions; and Phase 2) all other people [5].

### Model scenarios

Vaccine delivery rates were assumed to be 10,000 vaccinations per day starting December 15th, 2020, targeting healthcare workers and nursing home populations, reaching ~ 65% of healthcare workers and nursing home populations within 1 month.

We assumed three alternative times for expanding vaccination beyond healthcare workers and nursing home residents: 4 weeks after vaccination launch (January 15th), 5 weeks after launch (January 21st), or 6 weeks after launch (February 1st). Vaccination rates were assumed to increase to 20,000, 50,000, 100,000, or 150,000 vaccinations per day for the first dose and to either prioritize residents age 65+ and essential workers as defined by Cybersecurity & Infrastructure Security Agency (CISA) [6], or to be applied homogenously without prioritization.

### Evaluation of hypotheses

We compared the number of infections, hospitalizations, and deaths between December 15th, 2020 and July 1st, 2021 under each scenario. We estimated the maximum prioritization bottleneck that would avert more deaths compared to vaccination without prioritization. In sensitivity analyses, we compared the effect of the emergence of a more transmissible variant on maximum prioritization bottleneck.

## Results

### Impact of vaccination expansion timing and speed

**Fig. 1:**
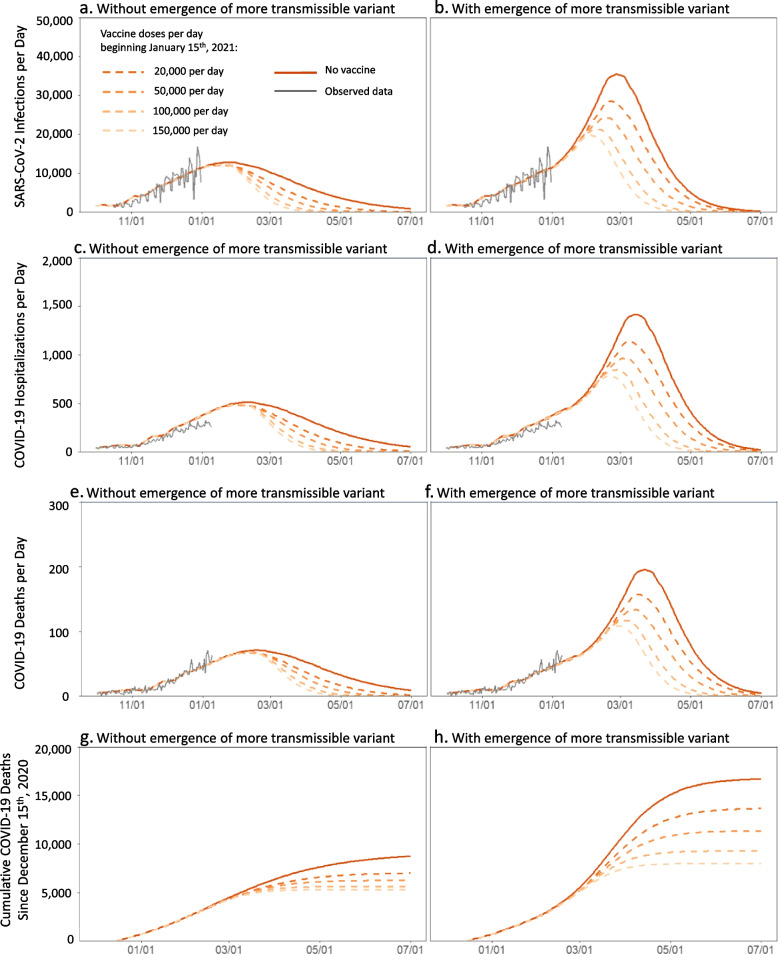
Daily new SARS-CoV-2 infections (**a**,**b**), hospitalizations (**c**,**d**), and deaths (**e**,**f**) as well as cumulative deaths since December 15th, 2020 (g,h) in NYC when achieving different vaccination speeds by January 15th, 2021. Results are computed using model scenarios without (**a**,**c**,**e**,**g**) and with (**b**,**d**,**f**,**h**) the emergence of a more transmissible SARS-CoV-2 variant, assumed to gradually increase in prevalence over the period of January 1st through February 28th, 2021 with a 56% increase in average SARS-CoV-2 transmissibility by the end of this period. For vaccines requiring multiple doses, the vaccination speeds reported are for the first dose only

**Fig. 2:**
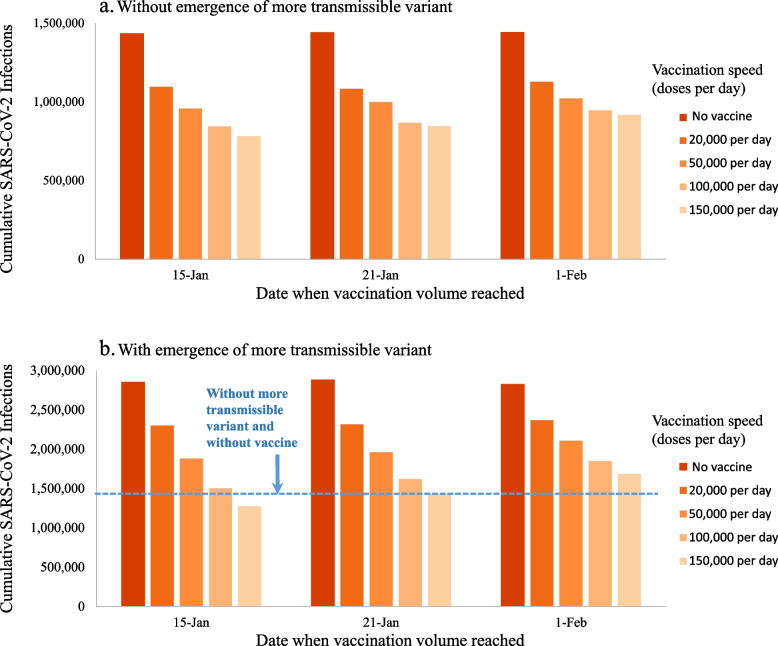
Cumulative SARS-CoV-2 infections over the period Dec 15th, 2020 to July 1st, 2021 when achieving different vaccination speeds by January 15th, January 21st, or February 1st, 2021. Results are computed using model scenarios without (**a**) and with (**b**) the emergence of a more transmissible SARS-CoV-2 variant, assumed to gradually increase in prevalence over the period of January 1st through February 28th, 2021 with a 56% increase in average SARS-CoV-2 transmissibility by the end of this period. For vaccines requiring multiple doses, the vaccination speeds reported are for the first dose only

Without vaccination, the COVID-19 pandemic was projected to cause 1,433,600 infections, 589,000 cases, 61,800 hospitalizations, and 8770 deaths in NYC between December 15th, 2020 and July 1st, 2021 (Fig. 1). At the slowest-examined vaccination speed of 20,000 vaccinations per day, expanding vaccine eligibility 4 weeks after launch would lower cumulative infections and deaths by 23.4% (from 1,433,600 to 1,095,300) and 20.0% (8770 to 7010) (Fig. 2). Delaying vaccine expansion to 6 weeks after launch would cause 31,500 (2.9%) additional infections and 179 (2.6%) additional deaths compared to immediate vaccine expansion. Compared to the slowest-examined vaccination speed of 20,000 vaccinations per day, achieving the fastest-examined vaccination speed of 150,000 vaccinations per day would avert additional 313,700 (28.6%) infections and 1693 (24.1%) deaths.

### Trade-off between vaccination speed and prioritization to high-risk groups

**Fig. 3:**
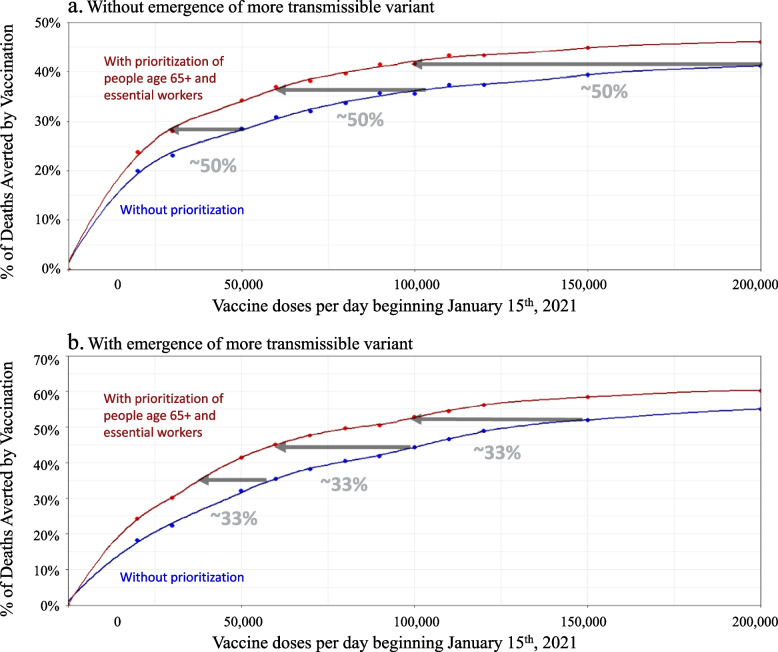
Percent of COVID-19 deaths averted over the period Dec 15th, 2020 to July 1st, 2021 by achieving different vaccination speeds by January 15th, 2021, compared to no vaccination beyond January 15th, 2021. Results are computed using model scenarios without (**a**) and with (**b**) the emergence of a more transmissible SARS-CoV-2 variant, assumed to gradually increase in prevalence over the period of January 1st through February 28th, 2021 with a 56% increase in average SARS-CoV-2 transmissibility by the end of this period. Red lines show deaths averted by vaccination with prioritization of individuals age 65+ and essential workers. Blue lines show deaths averted by vaccination without prioritization. Gray arrows demonstrate the prioritization bottleneck above which prioritization would no longer be favorable in terms of averting the most deaths. Dots show results of model runs, while lines show the cubic interpolation that was used to estimate the impact of vaccination speeds in-between the values directly evaluated in the model

Prioritizing age 65+ and essential workers averts more cumulative infections and deaths per vaccine dose delivered, as compared to vaccinating uniformly irrespective of priority groups. Saving 3000 lives – nearly one in 3000 New Yorkers – would require 83,000 vaccinations per day without prioritization but only 50,000 vaccinations per day with prioritization (Table 1). However, if prioritization were to reduce vaccination speed by causing an implementation bottleneck, the benefits would be reduced substantially. Prioritization would be beneficial in terms of deaths averted only if any resulting bottlenecks slowed vaccination speed by less than 50% (Fig. 3a).

**Table 1:** Vaccination speed (vaccinations per day beginning on January 15th, 2021) needed to avert different numbers of deaths in NYC over the period Dec 15th, 2020 to July 1st, 2021. For vaccines requiring multiple doses, the vaccination speeds reported are for the first dose only

Deaths averted	Without new variant		With new variant	
	No prioritization	Prioritization	No prioritization	Prioritization
2000	29,000	18,000	11,000	8,000
2500	50,000	31,000	15,000	10,000
3000	83,000	50,000	19,000	13,000
3500	163,000	81,000	27,000	16,000
4000	> 200,000	175,000	34,000	20,000
6000	> 200,000	> 200,000	61,000	40,000
8000	> 200,000	> 200,000	115,000	71,000

### Impact of a more transmissible variant on vaccination effects

The emergence of a variant with 56% greater transmissibility would increase by 3-fold the peak in daily COVID-19 infections, hospitalizations, and deaths in NYC in the absence of vaccination (Fig. 1a-f) and would add 7,947 COVID-19 deaths between December 15th, 2020 and July 1st, 2021, compared to without the emergence of a variant (Fig. 1g-h) – doubling the COVID-19 death toll over this period. Vaccine expansion concurrently with emergence of a more transmissible variant could substantially reduce the infections, hospitalizations, and deaths caused by the variant (Fig. 1). With expanded eligibility 4 weeks after vaccination launch, the slowest-examined vaccination speed of 20,000 vaccinations per day would reduce deaths added by the variant from 7,947 to 6,649 (Fig. 2). The highest-examined vaccination speed of 150,000 vaccinations per day would reduce deaths added by the variant further to 2,351, fewer than the deaths that would be averted by similarly-paced vaccination in the absence of the new variant (3,596). A vaccination speed of 120,000 or more vaccinations per day could offset the harm of the more transmissible variant.

In scenarios where vaccination expansion was delayed until 5 or 6 weeks after vaccination launch (Fig. 2b), the benefits of vaccination were smaller overall and required greater speeds to offset the harm of the more transmissible variant. The later the increase in vaccination speed, the smaller the benefit of achieving that speed. At 5 weeks post-launch, delivering 150,000 vaccinations per day would be required to offset the harm of a more transmissible variant, compared to 120,000 vaccinations with immediate vaccine expansion. At 6 weeks post-launch, delivering 150,000 vaccinations per day would not be sufficient to offset the harm of the more transmissible variant. Prioritization would only avert more deaths if any resulting bottlenecks slowed vaccination speed by no more than 33% (Fig. 3b).

### Prospective validation

We examined data on the proportion of circulating virus belonging to the B.1.1.7 lineage and the number of vaccines administered, cases, hospitalizations, and deaths, which became available after our modeling was conducted. The proportion of circulating virus belonging to the B.1.1.7 lineage reached a maximum of 49% by mid-May, 2021. Between December 15th, 2020 and July 1st, 2021, 4,778,161 individuals received at least one dose of vaccination, resulting in the average daily vaccination of 24,061 per day. The cumulative number of cases, hospitalizations, and deaths observed was 576,336, 41,916, and 8585, respectively, which fell in-between the model projections for 0% B.1.1.7 and 100% B.1.1.7. at similar vaccination rates (20,000 daily vaccinations together with prioritizing individuals age 65+ and essential workers), indicating consistency between our projections and prospective observations.

## Discussion

Our modeling results suggest that rapidly reaching 150,000 vaccinations per day could halve additional infections and deaths from the COVID-19 pandemic. Without the emergence of a more transmissible variant, prioritizing individuals age 65+ and essential workers could avert more deaths even if prioritization-induced bottlenecks reduced vaccination speed by up to half. However, with the variant, prioritizing could only avert more deaths if resulting bottlenecks reduced vaccination by up to one-third. Similar trade-offs between vaccination speed and prioritization were robust over a wide range of delivery capacity assumptions. With the emergence of a more transmissible variant, reaching at least 120,000 vaccinations per day would be required to offset the harm of the variant, and this speed would need to be reached within 4 weeks of vaccination launch.

Several modeling studies across the globe have estimated that highly effective vaccination with moderate to high coverage would be able to suppress the COVID-19 pandemic [25, 26]. Starting in May 2021, all adults have been eligible for COVID-19 vaccination in NYC [4]. Although some vaccination bottlenecks were initially observed, the daily doses administered reached ~ 50,000 per day, and 45% of NYC residents received at least the first dose by the end of April 2021 [4]. Our model results suggest that prioritization of high-risk groups, who are disproportionately affected by COVID-19 including older people and those with underlying conditions [1–3], likely contributed to averting infections, hospitalizations, and deaths during this period, although the true extent to which prioritization caused bottlenecks in vaccination speed are not well-measured.

By mid-May 2021, the lineage B.1.1.7 accounted for 49% of all sequenced cases in NYC, never fully dominating the mix of circulating variants [27]. The observed number of cumulative deaths during the first half of 2021 were between what we projected with and without B.1.1.7 emergence, for the scenario that best approximated the true vaccination rate in NYC (20,000 daily vaccinations together with prioritizing individuals age 65+ and essential workers). Based on these prospectively validated projections, in the absence of a vaccine and without substantial behavioral changes, the rapid growth of B.1.1.7 or other more transmissible variants would have tripled the 2021 epidemic peak and more than doubled COVID-19 burden.

Our analysis has several important limitations. The model did not stratify SARS-CoV-2 transmission patterns according to age, occupation, or neighborhood structure, nor did it incorporate the tendency for a relatively small proportion of individuals to produce a disproportionately large number of secondary cases – a phenomenon known as superspreading. Additionally, our simulations did not consider differences in pathogenicity among different variants, nor immune evasion by new variants. 

These limitations may bias our results in different ways. If prioritized occupations such as healthcare, education, and transit contribute more to SARS-CoV-2 transmission than other population groups, then our results may be biased in favor of vaccination speed rather than prioritization by failing to account for the transmission benefit of prioritizing essential workers. On the other hand, if even more transmissible variants than the B.1.1.7 lineage dominate in NYC, or if new variants are capable of re-infecting recovered individuals by evading naturally acquired immunity, our results may be biased in favor of prioritization rather than vaccination speed.

## Conclusions

Our modeling emphasizes that prioritization of COVID-19 vaccines to higher-risk populations saves more lives only if it does not create an excessive vaccine delivery bottleneck, as well as the urgency of rapid, high-speed vaccination in the context of the emergence of a more transmissible pathogen variant.

## Supplementary Information

**Additional file 1.**

## Data Availability

The data used are publicly available (https://github.com/nychealth/).

## Abbreviations

CDC: Centers for Disease Control and Prevention
CISA: Cybersecurity & Infrastructure Security Agency
COVID-19: Coronavirus Disease 2019
DOHMH: Department of Health and Mental Hygiene
HANES: Health and Nutrition Examination Survey
HCW: Healthcare workers
HERDS: Hospital Emergency Response Data System
NYC: New York City
SARS-CoV-2: Severe Acute Respiratory Syndrome Coronavirus 2
SEIR: Susceptible-Exposed-Infected-Recovered
UK: United Kingdom

## References

[1] Li J, Huang DQ, Zou B (2021). Epidemiology of COVID-19: a systematic review and meta-analysis of clinical characteristics, risk factors, and outcomes. J Med Virol.

[2] Parohan M, Yaghoubi S, Seraji A, Javanbakht MH, Sarraf P, Djalali M (2020). Risk factors for mortality in patients with coronavirus disease 2019 (COVID-19) infection: a systematic review and meta-analysis of observational studies. The Aging Male.

[3] Dessie ZG, Zewotir T (2021). Mortality-related risk factors of COVID-19: a systematic review and meta-analysis of 42 studies and 423,117 patients. BMC Infect Dis.

[4] New York City Department of Health and Mental Hygiene. COVID-19 Vaccination Reporting. https://github.com/nychealth/covid-vaccine-data. Accessed 8 June 2021.

[5] Dooling K. The advisory committee on immunization practices’ updated interim recommendation for allocation of COVID-19 vaccine — United States, December 2020. MMWR Morb Mortal Wkly Rep. 2021;69. 10.15585/mmwr.mm695152e2. 10.15585/mmwr.mm695152e2 PMC9191902 33382671

[6] CISA Releases Updated Guidance on Essential Critical Infrastructure Workers | CISA. https://www.cisa.gov/news/2020/08/18/cisa-releases-updated-guidance-essential-critical-infrastructure-workers. Accessed 26 Jan 2021.

[7] Board TE. Opinion | We Came All This Way to Let Vaccines Go Bad in the Freezer? The New York Times. https://www.nytimes.com/2020/12/31/opinion/coronavirus-vaccines-expiring.html. Published December 31, 2020. Accessed 26 Jan 2021.

[8] Stacey K. US states urged to be more flexible distributing Covid-19 jabs. Published January 6, 2021. https://www.ft.com/content/e3758509-7b5d-40e9-ba4c-46dedaf87968. Accessed 26 Jan 2021.

[9] Volz E, Mishra S, Chand M (2021). Assessing transmissibility of SARS-CoV-2 lineage B.1.1.7 in England. Nature.

[10] Volz E, Mishra S, Chand M, et al. Transmission of SARS-CoV-2 lineage B.1.1.7 in England: insights from linking epidemiological and genetic data. medRxiv. 2021:2020.12.30.20249034. 10.1101/2020.12.30.20249034.

[11] Wu JT, Leung K, Leung GM (2020). Nowcasting and forecasting the potential domestic and international spread of the 2019-nCoV outbreak originating in Wuhan, China: a modelling study. Lancet.

[12] Thakkar N, Selvaraj P, Famulare M, Klein D. *COVID in New York City: A Model-Based Perspective*.; 2020. https://covid.idmod.org/

[13] Kim HY, McGillen JB, Bershteyn A, Braithwaite RS (2020). Requirements for testing, tracing, and quarantine to enable safe relaxation of COVID-19 stay-at-home policies in a US metropolitan area: a mathematical modelling analysis. 23rd international AIDS conference.

[14] Centers for Disease Control and Prevention (2017). Behavioral risk factor surveillance system survey data.

[15] Thorpe LE, Greene C, Freeman A (2015). Rationale, design and respondent characteristics of the 2013–2014 new York City health and nutrition examination survey (NYC HANES 2013–2014). Prev Med Rep.

[16] United States Census Bureau (2018). 2013–2017 American community survey 5-year estimates.

[17] Shah K, Saxena D, Mavalankar D (2020). Secondary attack rate of COVID-19 in household contacts: a systematic review. QJM.

[18] Lauer SA, Grantz KH, Bi Q, et al. The incubation period of coronavirus disease 2019 (COVID-19) from publicly reported confirmed cases: estimation and application. Ann Intern Med. 2020. 10.7326/M20-0504. 10.7326/M20-0504 PMC7081172 32150748

[19] Cummings MJ, Baldwin MR, Abrams D (2020). Epidemiology, clinical course, and outcomes of critically ill adults with COVID-19 in new York City: a prospective cohort study. Lancet.

[20] New York City Department of Health and Mental Hygiene. NYC Coronavirus Disease 2019 (COVID-19) Data. Published 2020. https://github.com/nychealth/coronavirus-data. Accessed 25 Jan 2021.

[21] Centers for Disease Control and Prevention (2020). Coronavirus disease 2019 (COVID-19) 2020 interim case definition. Approved April 5, 2020.

[22] Thompson CN. COVID-19 outbreak — new York City, February 29–June 1, 2020. MMWR Morb Mortal Wkly Rep. 2020;69. 10.15585/mmwr.mm6946a2. 10.15585/mmwr.mm6946a2 PMC7676643 33211680

[23] Polack FP, Thomas SJ, Kitchin N (2020). Safety and efficacy of the BNT162b2 mRNA Covid-19 vaccine. N Engl J Med.

[24] Cristian Acero, Hilda Razzaghi, Carla L. Black, et al. Influenza Vaccination Coverage Among Health Care Personnel - United States, 2019–2020 Influenza Season. Published October 1, 2020. https://www.cdc.gov/flu/fluvaxview/hcp-coverage_1920estimates.htm. Accessed 9 Nov 2022.

[25] Moore S, Hill EM, Dyson L, Tildesley MJ, Keeling MJ (2021). Modelling optimal vaccination strategy for SARS-CoV-2 in the UK. PLoS Comput Biol.

[26] Iboi EA, Ngonghala CN, Gumel AB (2020). Will an imperfect vaccine curtail the COVID-19 pandemic in the U.S.?. Infect Dis Model.

[27] New York City Department of Health and Mental Hygiene. COVID-19: Data on Variants. https://www1.nyc.gov/site/doh/covid/covid-19-data-variants.page. Accessed 11 June 2021.

