# Supplementary material for: How severe would prioritization-induced bottlenecks need to be offset the benefits from prioritizing COVID-19 vaccination to those most at risk in New York City?
Source: BMC Public Health. 2023 Jan 26;23:174. doi: 10.1186/s12889-022-14846-7 (PMC9876757; doi:10.1186/s12889-022-14846-7)
Supplement: Supplementary file 1 — Additional file 1. [file 12889_2022_14846_MOESM1_ESM.docx]

**Supplementary material**

**How severe would prioritization-induced bottlenecks need to be offset the benefits from prioritizing COVID-19 vaccination to those most at risk?**

**SEIR transmission model**

We adapted an existing stochastic, discrete-time compartment model of community transmission of SARS-CoV-2 based on the paper by Wu *et al*. (1) and was previously described by Thakkar *et al.*(2). The model structures can be described as following:

$$S_{t}=S_{t-1}- \beta S_{t-1}\left( I_{t-1}+z_{t-1} \right)\varepsilon_{t}$$

$$E_{t}=\beta S_{t-1}\left( I_{t-1}+z_{t-1} \right)\varepsilon_{t}+\left( 1-\frac{1}{D_{e}} \right)E_{t-1}$$

$$I_{t}=\frac{1}{D_{e}}E_{t-1}+ \left( 1-\frac{1}{D_{i}} \right)I_{t-1}$$

$$R_{t}= \frac{1}{D_{I}}I_{t-1}$$

$$C_{t} \sim Binomial\left( I_{t}, p \right)$$

where 𝐷_𝑒_ is the latency period (4 days), 𝐷_𝑖_ is the infectious duration (8 days), 𝛽 is the attack rate at the beginning of the outbreak, and 𝑧_t_ is the number of importations on day 𝑡. In New York City (NYC), 𝑧_t_  was set to 7 on January 10^th^, 2020 for the importations linked to Wuhan, and 7 on February 22^nd^, 2020 for the cases linked to other global importations. In the model, transmission is a log-normal stochastic process with Var[ln 𝜀_t_] = 𝜎_𝜀_^2^ while case detection is a binomial process with reporting rate 𝑝. We have set 𝜎_𝜀_ to 0.722, a value determined from Seattle’s 2018-19 influenza season.

**Model input parameters and assumptions**

The model accounted for age-specific and comorbidity-adjusted infection-fatality ratio (IFR). We calculated the age-specific IFR adjusting for the increased severity for hospitalizations and deaths among those with underlying comorbidities by age groups (10-19; 20-29; 30-39; 40-49; 50-59; 60-69; 70-79; 80+ years). The increased risk of severe illness associated among COVID-19 infected patients with underlying comorbidity conditions was inferred from the report by Guan et *al* (3) during the early COVID-19 epidemic in China (Table 1). IFR was inferred from the case-fatality ratio in South Korea by mid-March 2020 (Table 2). Given that the testing and contact tracing were widely conducted in Korea, we assumed that CFR would provide a reasonable approximation for IFR. By early March, ~95% of the contacts of the confirmed index cases related to the main outbreak cluster in Daegu, Korea, which accounted for >79% all confirmed cases in Korea, were traced and tested (4). Therefore, we assumed the IFR would be slightly lower by 5%, resulting in the overall IFR estimate of 0.94. Then, we estimated the IFR in NYC adjusting for the age and comorbidities distribution in NYC obtained from New York Behavioral Risk Factor Surveillance System Data in 2017(5) and census(6) (Table 3). The model estimated the impact of the epidemic on COVID-19 hospitalization and mortality using time-varying values for the epidemic and disease progression from March 2020 to December 2020 in NYC (Table 4).

**Table S1. Risk of composite endpoints (admission to intensive care unit, invasive ventilation, or death) among 1,590 patients with COVID-19 in China by comorbidity condition in Guan et *al.* study (3).**

|  | Risk Ratio* | 95% CI |
| --- | --- | --- |
| **Comorbidity condition** |  |  |
| Diabetes | 1.586 | 1.028 - 2.449 |
| Hypertension | 1.575 | 1.069 - 2.322 |
| Chronic obstructive pulmonary disease (COPD) | 2.681 | 1.424 - 5.048 |
| Malignancy | 3.501 | 1.604 - 7.643 |
| **Number of comorbidity conditions** | |  |
| 1 | 1.789 | 1.424 - 5.048 |
| 2+ | 2.592 | 1.611 - 4.171 |

*The model was adjusted with age and smoking status.

**Table S2. COVID-19 cases and deaths in South Korea as of March 17, 2020, and the estimated age-specific infection-fatality rate (IFR) among those without two major comorbidity conditions (COPD and diabetes)**

| Age groups (years) | Number of confirmed cases(7) | Number of deaths | Case-fatality rate, % | Male population, 2016(8) | Female population, 2016 | Prevalence of COPD, 2016(9) | Prevalence of diabetes, 2016(9) | Age-specific IFR among those without COPD and diabetes, %** |
| --- | --- | --- | --- | --- | --- | --- | --- | --- |
| 10-19 | 522 | 0 | 0.00 | 2,478,773 | 2,314,563 | - | - | 0.01 |
| 20-29 | 2330 | 0 | 0.00 | 3,573,667 | 3,232,486 | - | - | 0.01 |
| 30-39 | 856 | 1 | 0.12 | 3,541,548 | 3,331,569 | - | 2.7% | 0.11 |
| 40-49 | 1164 | 1 | 0.09 | 4,214,971 | 4,079,816 | 4.0% | 8.0% | 0.07 |
| 50-59 | 1602 | 6 | 0.37 | 4,362,544 | 4,282,470 | 8.8% | 14.2% | 0.28 |
| 60-69 | 1033 | 15 | 1.45 | 3,298,604 | 3,445,902 | 21.6% | 21.8% | 0.85 |
| 70-79 | 539 | 30 | 5.57 | 1,664,340 | 2,033,906 | 32.8% | 28.9% | 2.75 |
| 80+ | 274 | 31 | 11.31 | 670,870 | 1,332,924 | 32.8% | 28.9% | 5.58 |
| Overall | 8320 | 84 | 1.01 | 23,805,317 | 24,053,636 |  |  |  |

*Age-specific IFR was calculated by adjusting the age-specific case-fatality rate for a factor of 0.94. For those aged 10-19 and 20-29 years, an insignificant number (0.01%) was added to avoid age-specific IFRs resulting in zero.

****** COPD: Chronic obstructive pulmonary disease

**Table S3. COVID-19 confirmed cases in New York City as of March 17, 2020, and the estimated age-adjusted and comorbidity-adjusted infection-fatality rate (IFR) by age groups**

|  | Values | Source |
| --- | --- | --- |
| **Distribution of confirmed COVID-19 cases in NYC by age groups (years)** | % |  |
| 0-19 | 4.4% |  |
| 20-29 | 15.9% |  |
| 30-39 | 15.9% | (10) |
| 40-49 | 16.7% |  |
| 50-59 | 17.5% |  |
| 60-69 | 14.8% |  |
| 70-79 | 9.3% |  |
| 80+ | 5.4% |  |
| **Population distribution by age groups (years)** | % |  |
| 0-9 | 12.2 | (6) |
| 10-19 | 11.0 |  |
| 20-29 | 16.4 |  |
| 30-39 | 15.7 |  |
| 40-49 | 13.0 |  |
| 50-59 | 12.5 |  |
| 60-69 | 9.9 |  |
| 70-79 | 5.6 |  |
| 80+ | 3.6 |  |
| **Prevalence of comorbidity conditions* by age groups (years)** |  |  |
| ***0 condition*** |  |  |
| 20-29 | 87.5% | (11) |
| 30-39 | 84.4% |  |
| 40-49 | 69.7% |  |
| 50-59 | 59.0% |  |
| 60-69 | 49.0% |  |
| 70-79 | 41.9% |  |
| 80+ | 27.5% |  |
| ***1 condition*** |  |  |
| 20-29 | 12.0% |  |
| 30-39 | 13.9% |  |
| 40-49 | 26.7% |  |
| 50-59 | 31.2% |  |
| 60-69 | 38.1% |  |
| 70-79 | 31.9% |  |
| 80+ | 43.4% |  |
| ***2+ conditions*** |  |  |
| 20-29 | 0.4% |  |
| 30-39 | 1.7% |  |
| 40-49 | 3.6% |  |
| 50-59 | 9.8% |  |
| 60-69 | 12.9% |  |
| 70-79 | 26.3% |  |
| 80+ | 29.1% |  |
| **Age-adjusted and comorbidity-adjusted IFR in NYC by age groups (years)** |  |  |
| 10-19 | 0.01% |  |
| 20-29 | 0.01% |  |
| 30-39 | 0.12% |  |
| 40-49 | 0.09% |  |
| 50-59 | 0.39% |  |
| 60-69 | 1.29% |  |
| 70-79 | 4.58% |  |
| 80+ | 10.08% |  |

*Chronic conditions included five self-reported conditions collected by the Behavioral Risk Factor Surveillance System from 2011 through 2016 for New York adults: cancer, chronic obstructive pulmonary disease (COPD), diabetes, heart disease, and high blood pressure.

**Table S4. Model input parameters for the epidemic and disease progression for COVID-19 in New York City**

| **Description** | **Values** | **Sources** |
| --- | --- | --- |
| **Epidemic and disease progression** |  |  |
| Proportion of asymptomatic infections | 60.0% | (12–14) |
| Proportion of symptomatic infections | 40.0% | (12–14) |
| Mild symptoms among symptomatic infections* | 73.5 – 90.5% | (10) |
| Severe symptoms among symptomatic infections* | 7.0 - 16.0% | (10) |
| Critical symptoms among symptomatic infections* |  |  |
| Critical ill patients admitted to hospitals among symptomatic infections | 2.5 - 9.0% | (10) |
| Critically ill patients outside the hospitals among symptomatic infections | 1.5% | (10) |
| Proportion of hospitalizations among the cases* | 9.5 - 26.6% | (10) |
| Proportion of ICU admission among the hospitalized* | 25.8 - 36.0% | (10); HERDS |
| Proportion of deaths among critically ill patients admitted to ICU* | 54.4 - 80.0% | (10) |
|  |  |  |
| ***Time to outcome (days)*** |  |  |
| Time to symptom onset (incubation period) | 5.1 | (15) |
| Time from symptom onset to hospitalization | 11.0 | (10) |
| Time from symptom onset to ICU hospitalization* | 14.0 | NYC DOHMH |
| Time from symptom onset to death* | 16.0 - 19.0 | NYC DOHMH |
| Time in hospitalization for severely ill patients* | 5.0 - 11.0 | NYC DOHMH |
| ICU length of stays for survival* | 6.0 - 21.0 | NYC DOHMH |
| Hospital length of stays* | 1.0 - 14.0 | NYC DOHMH |
|  |  |  |
| Case-fatality-ratio by age groups |  |  |
| 65+ years | 4.5% | (10) |
| <65 years | 0.4% | (10) |

*Time-varying values from March 2020 to December 2020 based on the information provided by NYC DOHMH

**We assumed that individuals with severe and critical symptoms would be hospitalized.

***HERDS: Hospital Emergency Response Data System; NYC DOHMH: New York City Department of Health and Mental Hygiene

**References**

1. Wu JT, Leung K, Leung GM. Nowcasting and forecasting the potential domestic and international spread of the 2019-nCoV outbreak originating in Wuhan, China: a modelling study. Lancet. 2020 Feb 29;395(10225):689–97.

2. Thakkar N, Selvaraj P, Famulare M, Klein D. COVID in New York City: A Model-Based Perspective [Internet]. 2020. Available from: https://covid.idmod.org/

3. Guan W jie, Liang W hua, Zhao Y, Liang H rui, Chen Z sheng, Li Y min, et al. Comorbidity and its impact on 1590 patients with COVID-19 in China: a nationwide analysis. Eur Respir J. 2020 May;55(5):2000547.

4. Daegu Disaster Management Headquater. COVID-19 daily briefing in Daegu metropolitan City (March 8th, 2020, 10:30am) [Internet]. 2020 [cited 2021 Jul 21]. Available from: https://blog.naver.com/daegu_news/221843112460

5. Centers for Disease Control and Prevention. Behavioral Risk Factor Surveillance System Survey Data. Atlanta, Georgia; 2017.

6. United States Census Bureau. 2013-2017 American Community Survey 5-Year Estimates. 2018.

7. Central Disaster Management Headquarters. Cases in Korea by City/Province [Internet]. 2020 [cited 2020 Apr 30]. Available from: http://ncov.mohw.go.kr/en/bdBoardList.do?brdId=16&brdGubun=162&dataGubun=&ncvContSeq=&contSeq=&board_id=&gubun=

8. Ministry of the Interior and Safety, the Government of South Korea. Demographics of registered residents [Internet]. 2020 [cited 2020 Mar 20]. Available from: https://jumin.mois.go.kr/

9. Korea Centers for Disease Control and Prevention. Korea National Health and Nutrition Examination Survey: 2016 Health Behavior and Chronic Disease Statistics [Internet]. Available from: https://knhanes.kdca.go.kr/knhanes/main.do

10. New York City Department of Health and Mental Hygiene. NYC Coronavirus Disease 2019 (COVID-19) Data [Internet]. 2020 [cited 2021 Jan 25]. Available from: https://github.com/nychealth/coronavirus-data

11. New York State Department of Health. Behavioral Risk Factor Surveillance System (BRFSS) Reports [Internet]. [cited 2020 Mar 25]. Available from: https://www.health.ny.gov/statistics/brfss/reports/

12. Mizumoto K, Kagaya K, Zarebski A, Chowell G. Estimating the asymptomatic proportion of coronavirus disease 2019 (COVID-19) cases on board the Diamond Princess cruise ship, Yokohama, Japan, 2020. Eurosurveillance [Internet]. 2020 Mar 12 [cited 2021 Jul 23];25(10). Available from: https://www.eurosurveillance.org/content/10.2807/1560-7917.ES.2020.25.10.2000180

13. Oran DP, Topol EJ. Prevalence of Asymptomatic SARS-CoV-2 Infection: A Narrative Review. Annals of Internal Medicine. 2020 Sep 1;173(5):362–7.

14. Wu Z, McGoogan JM. Characteristics of and important lessons from the Coronavirus disease 2019 (COVID-19) outbreak in China: summary of a report of 72 314 cases from the Chinese Center for Disease Control and Prevention. JAMA. 2020;323(13):1239.

15. Lauer SA, Grantz KH, Bi Q, Jones FK, Zheng Q, Meredith HR, et al. The Incubation Period of Coronavirus Disease 2019 (COVID-19) From Publicly Reported Confirmed Cases: Estimation and Application. Annals of Internal Medicine. 2020 May 5;172(9):577–82.
